# Supplementary material for: Mutations in PCYT2 disrupt etherlipid biosynthesis and cause a complex hereditary spastic paraplegia
Source: Brain. 2019 Oct 22;142(11):3382–97. doi: 10.1093/brain/awz291 (PMC6821184; doi:10.1093/brain/awz291)
Supplement: awz291_Supplementary_Data [file awz291_supplementary_data.zip › awz291-Suppl_data/Supplementary_Data4.zip!/Supplementary information 3 - fibroblast lipidomics.zip!/Fibroblast lipidomics/Controle vs Patient2/Statistical plots and data/Volcano plot_alternative.pdf]

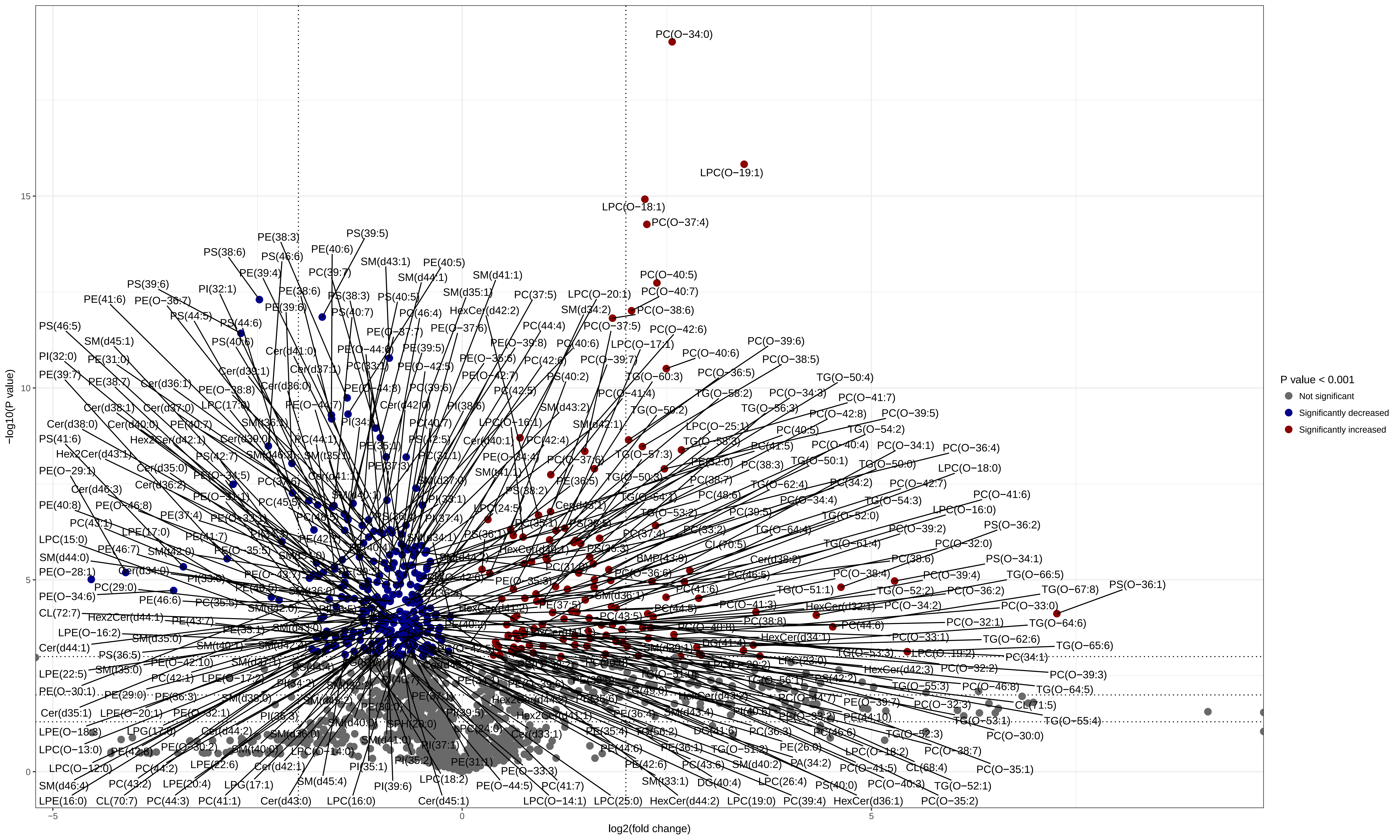

−log10(P value)
log2(fold change)
−5
0
5
15
10
5
0
P value < 0.001
Not significant
Significantly decreased
Significantly increased
PC(O−34:0)
LPC(O−19:1)
LPC(O−18:1)
PC(O−37:4)
PC(O−40:5)
PC(O−40:7)
PC(O−38:6)
PC(O−42:6)
PC(O−39:6)
PC(O−38:5)
PC(O−40:6)
PC(O−36:5)
TG(O−50:4)
PC(O−34:3)
PC(O−41:7)
PC(O−42:8)
PC(O−39:5)
TG(O−54:2)
PC(O−40:4)
PC(O−34:1)
PC(O−36:4)
LPC(O−18:0)
PC(O−42:7)
PC(O−41:6)
LPC(O−16:0)
PS(O−36:2)
PC(O−39:2)
PC(O−32:0)
PS(O−34:1)
TG(O−66:5)
TG(O−67:8)
PS(O−36:1)
PC(O−33:0)
TG(O−64:6)
PC(O−32:1)
PC(O−33:1)
TG(O−62:6)
TG(O−65:6)
PC(34:1)
PC(O−39:3)
TG(O−64:5)
CL(71:5)
TG(O−55:4)
PC(O−30:0)
PC(O−38:7)
PC(O−35:1)
TG(O−52:1)
PC(O−35:2)
PS(39:5)
PE(38:3)
PE(40:6)
PS(38:6)
PS(46:6)
PE(39:4)
SM(d43:1)
PE(40:5)
SM(d44:1)
SM(d41:1)
PS(39:6)
PI(32:1)
PE(41:6)
PE(O−36:7)
PS(44:5)
PS(46:5)
SM(d45:1)
PI(32:0)
PE(31:0)
PE(39:7)
PE(38:7)
Cer(d36:1)
Cer(d38:1)
Cer(d37:0)
Cer(d38:0)
Cer(d40:0)
PE(40:7)
PS(41:6)
Hex2Cer(d42:1)
Hex2Cer(d43:1)
PE(O−29:1)
Cer(d46:3)
PE(40:8)
PE(O−46:8)
PC(43:1)
PE(37:4)
LPC(15:0)
LPE(17:0)
PE(46:7)
SM(d42:0)
SM(d44:0)
Cer(d34:0)
PE(O−28:1)
PC(29:0)
PE(O−34:6)
PE(46:6)
CL(72:7)
Hex2Cer(d44:1)
LPE(O−16:2)
Cer(d44:1)
PS(36:5)
SM(d35:0)
LPE(22:5)
SM(t35:0)
PE(O−30:1)
PE(29:0)
Cer(d35:1)
LPE(O−20:1)
LPE(O−18:3)
LPG(17:0)
LPC(O−13:0)
PE(42:8)
LPC(O−12:0)
PC(44:2)
SM(d46:4)
PC(43:2)
LPE(16:0)
CL(70:7)
PC(44:3)
PC(41:1)
Cer(d43:0)
LPC(16:0)
Cer(d45:1)
LPC(O−14:1)
LPC(25:0)
HexCer(d44:2)
LPC(19:0)
PC(39:4)
HexCer(d36:1)
PI(39:6)
LPC(18:2)
PE(O−44:5)
PC(41:7)
SM(d45:4)
LPG(17:1)
PE(20:4)
LPE(22:6)
Cer(d42:1)
PI(35:1)
PI(35:2)
PE(31:1)
PE(O−33:3)
SM(t33:1)
DG(40:4)
LPC(26:4)
PS(40:0)
PC(O−40:3)
PC(O−41:5)
CL(68:4)
LPC(O−18:2)
PA(34:2)
PE(26:0)
SM(d40:2)
PC(43:6)
PE(42:6)
PE(36:1)
PE(44:6)
TG(O−51:2)
